# Background qualitative analysis of the European reference life cycle database (ELCD) energy datasets – part II: electricity datasets

**DOI:** 10.1186/s40064-015-0812-2

**Published:** 2015-01-23

**Authors:** Daniel Garraín, Simone Fazio, Cristina de la Rúa, Marco Recchioni, Yolanda Lechón, Fabrice Mathieux

**Affiliations:** CIEMAT, Energy Department, Energy Systems Analysis Unit, Av. Complutense 40, Madrid, E28040 Spain; European Commission, Joint Research Centre, Institute for Environment and Sustainability, Unit JRC.H.8-Sustainability Assessment, Via E. Fermi 2749, TP 270-21027 Ispra, Italy

**Keywords:** ELCD, Electricity, ILCD, Data Quality

## Abstract

The aim of this paper is to identify areas of potential improvement of the European Reference Life Cycle Database (ELCD) electricity datasets. The revision is based on the data quality indicators described by the International Life Cycle Data system (ILCD) Handbook, applied on sectorial basis. These indicators evaluate the technological, geographical and time-related representativeness of the dataset and the appropriateness in terms of completeness, precision and methodology. Results show that ELCD electricity datasets have a very good quality in general terms, nevertheless some findings and recommendations in order to improve the quality of Life-Cycle Inventories have been derived. Moreover, these results ensure the quality of the electricity-related datasets to any LCA practitioner, and provide insights related to the limitations and assumptions underlying in the datasets modelling. Giving this information, the LCA practitioner will be able to decide whether the use of the ELCD electricity datasets is appropriate based on the goal and scope of the analysis to be conducted. The methodological approach would be also useful for dataset developers and reviewers, in order to improve the overall Data Quality Requirements of databases.

## Introduction

The European Platform of Life Cycle Assessment (EPLCA), a project initiated by the Institute for Environment and Sustainability (IES), has the objective of promoting Life Cycle Thinking (LCT) and providing appropriate support to business and public administrations within the European Union (EU), as well as in close coordination with international activities. This support is essential, and is being achieved through the development of a number of different deliverables, being the European Reference Life Cycle Database (ELCD) one of them. The ELCD provides core Life Cycle Inventory (LCI) data from front-running EU-level business associations and, where not available, other sources. Several energy-related data are provided within the ELCD, since energy is a key input to most environmental analyses of products or processes. The ELCD latest version can be consulted on the JRC webpage: http://eplca.jrc.ec.europa.eu/ELCD3/.

Data quality in LCA still represents a major bottleneck to a broader use of LCA and environmental footprint methods in business and in policy (Fazio et al. Method applied to the background analysis of energy data to be considered for the European Reference Life Cycle Database (ELCD). Springer Plus – Submitted in 2014). Under the framework of ISO standards (ISO 14044:2006) some guidelines have been developed to address several requirements: ILCD handbook, UNEP/SETAC LC Initiative, or USLCI Database (Garraín et al. Background qualitative analysis of the European Reference Life Cycle Database (ELCD) energy datasets – Part I: Fuel datasets. Springer Plus - Submitted in 2014).

The objective of this analysis is to identify areas of potential improvement of the ELCD electricity datasets quality, considering data available in third party life cycle databases and from authoritative bodies and/or business associations. The work has consisted in analysing and comparing electricity datasets from different databases, considering the ELCD database as the basis for this analysis. This effort has been carried out in two stages: i) Selection of datasets, databases and quality standards, in order to assure the methodology, ii) Analysis and qualitative comparison of the datasets, each selected electricity dataset was analysed according to previously defined quality standards. Then, findings and recommendations were derived in order to identify the potential improvements of ELCD datasets.

## Methodology

### Selection of datasets and databases

The energy datasets to be analysed should be representative of a significant share (such as 40 to 60%) of the European electricity market and associated technology mixes/geographic origins, therefore a deep review of the most updated data in terms of electricity for EU-27 has been conducted.

According to European statistics (EUROSTAT, 2012; EC, 2011), the energy sources that contribute the most to electricity generation in 2011 were the following: Nuclear (27%), coal (26%), gas (23%), hydro (13%) and wind (4%). Other renewable energy sources have lower contribution to electricity generation in EU-27, such as biomass and waste, and solar energy (3% and 0.68%, respectively). However, due to their foreseen potentials, their contribution is expected to increase in the future. So, electricity from these sources was considered for the analysis. An electricity mix for EU-27 was also taken into account.

The latest ELCD version includes one dataset of European average electricity mix as well as electricity mix datasets from each EU-27 country. However, the unit processes used to build the datasets cannot be broken down into technologies. This limitation had to be solved, since the final objective of the analysis is to analyse the quality of the different datasets, focusing on the underlying models and data used. These ELCD electricity mix datasets by country have been originated from PE International (GaBi 2012). Taking into account the above mentioned limitation, the use of specific datasets from GaBi for conducting the analysis seemed to be essential. Whenever ELCD database did not provide the required datasets, GaBi datasets from the last updated version were analysed. It must be noticed that GaBi provides these datasets for each EU-27 country, but does not include datasets for each technology referring to the European context (which are available in the developer’s internal database -PE International-, but so far not in the commercially available databases, *i.e.* electricity production from hard coal, European Mix). As a first approximation, in order to take into account the European energy market, the datasets by country were chosen from GaBi database considering only those countries that sum up 60% of the electricity produced in Europe for each technology (this value has been decided by the leaders of this evaluation as a first approach, and considering that it will be representative enough for the European energy market). Hereinafter, the nomenclature of ELCD energy datasets will refer to GaBi datasets.

In order to identify those countries that sum up more than 60% of the electricity produced in Europe by technology, data of electricity production by sources from Eurostat (data from 2010) were collected and analysed. Germany (23%), United Kingdom (21%) and Poland (20%) were the main producers of electricity from hard coal; Germany (41%), Czech Republic (14%), Poland (14%) and Greece (9%) were the main contributors to lignite electricity production; the main producers of electricity from natural gas were United Kingdom (20%), Italy (20%), Germany (13%) and Spain (10%); and the main producers of electricity from nuclear power were France (47%) and Germany (15%). Then, Table 1 shows the eighteen chosen datasets as the base for the comparison with other datasets.

**Table 1:** **List of the selected ELCD electricity datasets as basis for comparison**

**Electricity from**	**Location**	**Name of LCI process/dataset**
Mix	EU-27	EU-27: Electricity grid mix (1 kV - 60 kV)
Coal	Germany	DE: Electricity from hard coal (1 kV - 60 kV)
	United Kingdom	GB: Electricity from hard coal (1 kV - 60 kV)
	Poland	PL: Electricity from hard coal (1 kV - 60 kV)
Lignite	Germany	DE: Electricity from lignite (1 kV - 60 kV)
	Greece	GR: Electricity from lignite (1 kV - 60 kV)
	Poland	PL: Electricity from lignite (1 kV - 60 kV)
	Czech Republic	CZ: Electricity from lignite (1 kV - 60 kV)
Natural gas	United Kingdom	GB: Electricity from natural gas (1 kV - 60 kV)
	Italy	IT: Electricity from natural gas (1 kV - 60 kV)
	Germany	DE: Electricity from natural gas (1 kV - 60 kV)
	Spain	ES: Electricity from natural gas (1 kV - 60 kV)
Nuclear power	France	FR: Electricity from nuclear (1 kV - 60 kV)
	Germany	DE: Electricity from nuclear (1 kV - 60 kV)
Hydropower	EU-27	EU-27: Electricity from hydro power (1 kV - 60 kV)
Wind power	European average	RER: Electricity from wind power (1 kV - 60 kV)
Biomass	Germany	DE: Electricity from biomass (solid) (1 kV - 60 kV)
Solar	Germany	DE: Electricity from photovoltaic (1 kV - 60 kV)

These datasets have been compared to their counterparts from three other databases: Ecoinvent v2.2 (Ecoinvent 2012), GEMIS 4.7 (GEMIS 2012), and E3 database (E3 2012). Considering theses databases and the availability of datasets, Table 2 presents the list of datasets to be finally analysed. The database selection have been made irrespective of the methodological compliance of the database/datasets with the ILCD quality criteria: it was indeed assumed that although other databases might have lower data quality rating (DQR) according to ILCD rules (because they were not specifically developed using these rules), datasets would represent interesting benchmarks and some improvement could be derived from the background analysis (Fazio et al. Method applied to the background analysis of energy data to be considered for the European Reference Life Cycle Database (ELCD). Springer Plus – Submitted in 2014).

**Table 2:** **Selected datasets to be analysed by database**

**ELCD**	**Ecoinvent v2.2**	**GEMIS 4.7**	**E3**
EU-27: Electricity grid mix	Electricity, medium voltage, production RER, at grid/RER	El-generation-mix-EU-27-2010 (PRIMES)	Electricity/Electricity-Mix-EU (10-20 kV-level)
DE: Electricity from hard coal	Electricity, hard coal, at power plant/DE	Coal-ST-DE-import-2005	Power Station/Hard Coal/ST/Germany
		Coal-ST-DE-2005	
GB: Electricity from hard coal	-	Coal-ST-UK-2005	-
PL: Electricity from hard coal	Electricity, hard coal, at power plant/PL	Coal-ST-PL-2005	-
DE: Electricity from lignite	Electricity, lignite, at power plant/DE	Lignite-ST-DE-2005 Rhine	Power Station/Lignite ST/Rhine GER
		Lignite-ST-DE-2005 Lausitz	Power Station/Lignite ST/Lausitz GER
			Power Station/Lignite ST CHP/Leipzig
GR: Electricity from lignite	Electricity, lignite, at power plant/GR	Lignite-ST-GR-2010	-
PL: Electricity from lignite	Electricity, lignite, at power plant/PL	Lignite-ST-PL-2010	-
CZ: Electricity from lignite	Electricity, lignite, at power plant/CZ	Lignite-ST-CZ-HU 4x200 2005	-
GB: Electricity from natural gas	Electricity, natural gas, at power plant/GB	Gas-CC-UK-2010	-
IT: Electricity from natural gas	Electricity, natural gas, at power plant/IT	Gas-CC-IT-2010	-
DE: Electricity from natural gas	Electricity, natural gas, at power plant/DE	Gas-CC-DE-2010	Power Station/NG/CCGT
ES: Electricity from natural gas	Electricity, natural gas, at power plant/ES	Gas-CC-ES-2010	-
FR: Electricity from nuclear	Electricity, nuclear, at power plant/FR	Nucler-powerplant-PWR-FR-2000	Power Station/Nuclear (DWR-F)
		Nucler-powerplant-PWR-FR-2010 (EPR)	
DE: Electricity from nuclear	Electricity, nuclear, at power plant/DE	Nucler-powerplant-PWR-DE-2005	Power Station/Nuclear/PWR-GER
EU-27: Electricity from hydro power	Electricity, hydropower, at run-of-river power plant/RER	Hydro-dam-big-generic	-
	Electricity, hydropower, at reservoir power plant/RER		
RER: Electricity from wind power	Electricity, at wind power plant/RER	Windfarm-big-generic	Power Station/Wind/on-shore/Enercon E-66/20.70 (Germany)
			Power Station/Wind/off-shore/Horns Rev
DE: Electricity from biomass (solid)	-	Biomass-ST-EU-2010	Power Station/Biomass/STCHP/Pfaffenhofen
DE: Electricity from photovoltaic	Electricity, production mix photovoltaic, at plant/DE	Solar-PV-mon-framed-with-rack-DE-2010	Power Station/Photovoltaic/multi crystalline (990 kWh)
		Solar-PV-multi-framed-with-rack-DE-2010	

### Quality criteria for analysis

The evaluation has been based on the quality indicators developed within the ILCD handbook (EC-JRC, 2010a, 2010b, 2011): Technological representativeness (TeR), Geographical representativeness (GR), Time-related representativeness (TiR), Completeness (C), Precision/Uncertainty (P) and Methodological appropriateness and consistency (M). Each of those has been evaluated according to the degree of accomplishment of the criterion (from 1 to 5), and an overall DQR of the datasets has been calculated by summing up the achieved quality rating for each of the quality criteria indicator, divided by the total number of considered indicators, as described in Garraín et al. Background qualitative analysis of the European Reference Life Cycle Database (ELCD) energy datasets – Part I: Fuel datasets. Springer Plus - Submitted in 2014.

The quality indicators described in the ILCD Handbook (EC-JRC, 2011) provide a general framework to evaluate datasets. When applying these indicators to specific sectorial datasets, it is necessary to redefine them based on the specific characteristics of the processes/technologies in order to identify key aspects. For this purpose, a deep pre-analysis of the technology situation was conducted, considering the European market context. The main features for assessing each criterion are similar to those described in Fazio et al. Method applied to the background analysis of energy data to be considered for the European Reference Life Cycle Database (ELCD). Springer Plus – Submitted in 2014 and Garraín et al. Background qualitative analysis of the European Reference Life Cycle Database (ELCD) energy datasets – Part I: Fuel datasets. Springer Plus - Submitted in 2014. Table 3 shows both quality criteria definitions and values considered.

**Table 3:** **Matrix for assessing LCI of electricity datasets**

**Quality indicator**	**Sub-quality parameters**	**Rating**				
		**1 (Very Good)**	**2 (Good)**	**3 (Fair)**	**4 (Poor)**	**5 (Very Poor)**
TeR	Expert judgement based on the consideration of a technology mix	Technology aspects have been modelled as the technology mix	Technology aspects are very similar to the technology mix	Technology aspects are similar to the technology mix	Technology aspects are different to the technology mix	Technology aspects are completely different to the technology mix, or tech not deployed
GR	Expert judgement based on geographical coverage of data	The list of countries is the same as the referenced countries	The list of countries is very similar to the referenced countries	The list of countries is slightly different from the referenced countries	The list of countries is significantly different from the referenced countries	The list of countries is totally different from the referenced countries
TiR	Expert judgement based on defined time on data inventory (±5 years)	All the data sources refer to the defined time	The majority of the data sources refer to the defined time	At least half of the data sources refer to the defined time	Less than half of the data sources refer to the defined time	None of the data sources refer to the defined time
C	Consideration of impact categories and share of elementary flows (to adjust the final rating)	15-16 considered impact categories	12-14 considered impact categories	8-11 considered impact categories	5-7 considered impact categories	<5 considered impact categories
P	Expert judgement based on the precision/uncertainty of data sources	Very low uncertainty and/or very high precision	Low uncertainty and/or high precision	Fair uncertainty and/or fair precision	High uncertainty and/or low precision	Very high uncertainty and/or very low precision
M	Definition of situation context and subsequent expert judgement of system boundaries, multi-functionality and EoL	Inclusion of all LCA stages (with the EoL stage). Consideration of allocation procedures. Completion in a very high degree	Inclusion of most relevant LCA stages. Consideration of allocation procedures. Completion in a high degree	Inclusion of a still sufficient LCA stages. Consideration of allocation procedures. Completion in a sufficient degree	Inclusion of a sufficient LCA stages. Consideration of allocation procedures. Completion in a low degree	No inclusion of sufficient LCA stages. No consideration of allocation procedures (multi-functionality has not been solved according to the situation context). Completion in a low degree

## Results

Table 4 shows the rates of the quality criteria assessment of the selected ELCD electricity datasets. Information contained in each dataset and additional confidential documents provided by the dataset developer (GaBi, 2012) were considered to define a final single value for each criterion. It should be noticed that only one dataset of each technology has been included in this article in order to show the full application of the evaluation method.

**Table 4:** **Quality criteria and DQR values of electricity ELCD datasets**

**Datasets**	**Database**	**DQI**	**Score**	**Short justification of DQI**	**DQR**
Electricity grid mix (EU27)	ELCD	TeR	1	Modelled as the EU27 technology mix. Each Member State modelled with the own technology mix	1.17
		GR	1	Modelled according the most updated EU27 country mix	
		TiR	1	Ref. year 2009, data from 2006-2010	
		C	1	100% of impact categories and 95% of reference flows covered	
		P	2	Sources from national statistics and IEA, relevant flows measured. Elementary flows are quantified	
		M	1	Cradle-to-grave, EoL included, exergetic and market value allocation	
	Ecoinvent	TeR	1	Modelled as the EU technology mix. Each country modelled with the own technology mix	1.92
		GR	2	EU27 are included except Baltic countries. Norway, Switzerland and countries of former state of Yugoslavia are included	
		TiR	2	Ref. year 2004, data from average production in 2000. Reference period 2000-2002, some references from ‘90s	
		C	1	100% of impact categories and 100% of reference flows covered	
		P	2	References from authoritative sources, but no info about emission factors or direct emissions	
		M	3	Cradle-to-grave, EoL not included, allocation only in wastes	
	GEMIS	TeR	1	Modelled as the EU27 technology mix	1.92
		GR	1	Modelled according the most updated EU27 country mix (2010)	
		TiR	2	Ref. year 2010, main data from 2010, some from 2003	
		C	2	75% of impact categories and 90% of reference flows covered	
		P	3	Sources are relevant, but no info about emission factors or direct emissions	
		M	3	Cradle-to-grave, EoL not included, allocation applied but not defined	
	E3	TeR	3	Modelled as the technology mix, but obsolete (1999)	3.17
		GR	4	Electricity mix from 1999 (EU-15)	
		TiR	2	Ref. year 1999, data from JEC (2007)	
		C	4	Less than 50% impact categories, 90% flows covered	
		P	3	Sources are relevant, but no info about emission factors or direct emissions	
		M	3	Cradle-to-grave, EoL not included, allocation applied but not defined (assumed as GEMIS)	
Electricity from hard coal (DE)	ELCD	TeR	1	Both electricity and CHP plants considered, use of technology mix	1.50
		GR	2	Domestic production and imports considered, but slightly differences in shares of each country with the reference	
		TiR	2	Ref. year 2009, data from 2006-2010. Some emission data from ‘90s	
		C	1	100% of impact categories and 96% of reference flows covered	
		P	2	References from relevant literature and authoritative sources. Some emission data from outdated and no German conditions studies	
		M	1	Cradle-to-grave, EoL included, exergetic and market value allocation	
	Ecoinvent	TeR	2	Modelled as an average plant in EU, in German conditions. Infrastructure based in 2 units from ‘80s	2.00
		GR	3	Import countries fulfilled, but the share differs to the value in 2000	
		TiR	2	Ref. year 1993-2000, data from 1991-2004 (mainly from ‘90s)	
		C	1	100% of impact categories and 100% of reference flows covered	
		P	2	Emission from calculated data from power plants (internal document)	
		M	2	Cradle-to-grave, EoL not included but info about treatment of outputs, energy content allocation included (only in hard coal coke)	
	GEMIS	TeR	3	Modelled as a single plant in Germany	2.50
		GR	3	Import countries are the same as the reference, but no share of domestic vs imported hard coal	
		TiR	2	Ref. year 2005, data from 2001-2009	
		C	2	75% of impact categories and 90% of reference flows covered	
		P	2	Sources are authoritative sources, but no info about emission factors or direct emissions	
		M	3	Cradle-to-grave, EoL not included, allocation applied but not defined	
	E3	TeR	3	Modelled as a single plant in Germany	3.67
		GR	4	Consideration of EU mix of hard coal in 2009 (very different from the German coal imports in 2005)	
		TiR	3	Ref. year 2005, data from 2001-2009 (plants and mining) and from ‘90s (statistical data)	
		C	4	Less than 50% impact categories, 90% flows covered	
		P	3	Sources are relevant, but no info about emission factors or direct emissions	
		M	5	Cradle-to-gate, EoL not included, allocation not defined (assumed as GEMIS)	
Electricity from lignite (DE)	ELCD	TeR	1	Both electricity and CHP plants considered, use of technology mix	1.33
		GR	1	Domestic production (only 0.02% imported) and most updated data	
		TiR	2	Ref. year 2009, data from 2006-2010. Some emission data from ‘90s	
		C	1	100% of impact categories and 96% of reference flows covered	
		P	2	References from relevant literature and authoritative sources. Some emission data from outdated and no German conditions studies	
		M	1	Cradle-to-grave, EoL included, exergetic and market value allocation	
	Ecoinvent	TeR	2	Modelled as an average plant in EU, in German conditions. Infrastructure based in 2 units from ‘80s	1.92
		GR	2	Average EU conditions (RER) in lignite mining and power plant	
		TiR	2	Ref. year 1993-2000 (technology) and 1980-1992 (plants), data from 1991-2004 (mainly from ‘90s)	
		C	1	100% of impact categories and 100% of reference flows covered	
		P	2	Emission from calculated data from power plants (internal document)	
		M	2	Cradle-to-grave, EoL not included but info about treatment of outputs, energy content allocation included	
	GEMIS	TeR	3	Modelled as single plants in Germany (only one of coal)	2.75
		GR	2	Domestic production of lignite, plants sited in Germany	
		TiR	3	Ref. year 2010, data from 2001-2009	
		C	2	75% of impact categories and 90% of reference flows covered	
		P	3	Data comes from Oko Institute reports, but no info about emission factors or direct emissions	
		M	3	Cradle-to-grave, EoL not included, allocation applied but not defined	
	E3	TeR	3	Modelled as singles plants in Germany	3.42
		GR	2	Domestic production of lignite, plants sited in Germany	
		TiR	3	Ref. year 2010, data from GEMIS and Ecoinvent (Lausitz plant); Ref. year 1994, data from 1992 (rest of plants)	
		C	4	Less than 50% impact categories, 90% flows covered	
		P	4	Sources are relevant (not authoritative sources), but no info about emission factors or direct emissions	
		M	4	Cradle-to-gate, EoL not included, allocation not defined (assumed as GEMIS)	
Electricity from natural gas (GB)	ELCD	TeR	1	Both electricity and CHP plants considered, use of technology mix	1.50
		GR	2	Domestic production and imports considered, but slightly differences in shares of each country with the reference	
		TiR	2	Ref. year 2009, data from 2006-2010. Some emission data from ‘90s	
		C	1	100% of impact categories and 96% of reference flows covered	
		P	2	References from relevant literature and authoritative sources. Some emission data from outdated studies	
		M	1	Cradle-to-grave, EoL included, exergetic and market value allocation	
	Ecoinvent	TeR	2	Modelled as an average plant in EU, based in a German CHP plant	2.17
		GR	4	Only domestic origin, when imports represent 40-50% of raw material in 2009	
		TiR	2	Ref. year ‘90s, data from ‘90s (statistical reports)	
		C	1	100% of impact categories and 100% of reference flows covered	
		P	2	Emission from calculated data from power plants (internal document) and authoritative sources (IEA)	
		M	2	Cradle-to-grave, EoL not included, energy content allocation included in CHPs	
	GEMIS	TeR	3	Modelled as a single plant	3.33
		GR	4	No info of plant location. Roughly 80% of country suppliers are considered. Important increase of Qatar imports not considered (2009-2011)	
		TiR	4	Ref. year 2010, data from 1994-2003. Data cannot be checked	
		C	2	75% of impact categories and 90% of reference flows covered	
		P	4	Data comes from Oko Institute reports, but no info about emission factors or direct emissions	
		M	3	Cradle-to-grave, EoL not included, allocation applied but not defined	
Electricity from nuclear (FR)	ELCD	TeR	1	Modelled as the French technology mix	1.83
		GR	2	Some activities of milling and reprocessing refers to US data	
		TiR	3	Some references are 20 years older than the ref. year (2009)	
		C	1	100% of impact categories and 100% of reference flows covered	
		P	2	Relevant flows measured, other flows taken from literature	
		M	2	Cradle-to-grave, EoL of intermediate activities is missing	
	Ecoinvent	TeR	2	Some data extrapolated from Swiss power plants	1.67
		GR	2	Infrastructure data from Swiss plants, only 1 uranium supplier	
		TiR	2	Ref. year 2002, relevant data are more updated than ELCD	
		C	1	100% of impact categories and 100% of reference flows covered	
		P	2	Relevant flows measured, other flows taken from literature	
		M	1	EoL and allocation also for sub-processes	
	GEMIS	TeR	2	Referred to French representative plants but not as a mix	3.08
		GR	4	Only the modeling of enrichment is correct	
		TiR	2	(depending on plant) literature comes from 5-15 years before	
		C	2	75% of impact categories, 90% of flows covered	
		P	4	Literature data and auto-estimated data	
		M	4	EoL not modeled, not including infrastructures.	
	E3	TeR	4	Considering a process scale instead of real plant	4.00
		GR	4	Only the modeling of enrichment is correct	
		TiR	3	Reference year 2000, data from 1994-99	
		C	4	Less than 50% impact categories, 90% flows covered	
		P	4	Literature data and auto-estimated data	
		M	5	Cradle to gate system, EoL and infrastructure lacking	
Electricity from hydro power (EU27)	ELCD	TeR	1	Modelled as the EU27 technology mix (run-of-river, storage and pump storage)	1.33
		GR	1	Modelled according the EU27 mix	
		TiR	1	Ref. year 2009, data from 2005-2010	
		C	1	94% of impact categories and 96% of reference flows covered	
		P	2	Data of technology issue from authoritative sources, and data of energy consumption and emissions from specific countries	
		M	2	Cradle-to-grave, EoL of some parts included, allocation not applied	
	Ecoinvent	TeR	3	Technologies from Swiss and Austrian plants (reservoir and run-of-river) which represent the 5^th^ and 6^th^ countries in the ranking of electricity generation in EU	2.50
		GR	3	Modelled as Swiss conditions, extrapolated to the average RER	
		TiR	3	Ref. year 1945-2000, data from 1960-2004	
		C	1	100% of impact categories and 100% of reference flows covered	
		P	3	Technology comes from relevant sources and emissions are extrapolated (main reference is an internal document)	
		M	2	Cradle to gate system, possibility of EoL is included (in case of dismantling), no info about allocation	
	GEMIS	TeR	4	Modelled as a generic dam plant	3.67
		GR	5	No definition of countries, defined as a ‘generic’ dataset. Several non-European countries are included	
		TiR	4	Ref. year 2000, data from ‘90s, which collected data from previous years	
		C	2	75% of impact categories, 90% of flows covered	
		P	4	Data comes from Oko Institute reports, but no info about emission factors or direct emissions	
		M	3	Cradle-to-grave, EoL not included, allocation might be appropriate but not defined	
Electricity from wind power (RER)	ELCD	TeR	1	Modelled the onshore and offshore wind technologies available at the commercial level in Europe	1.17
		GR	1	Modelled for RER. Most relevant data related to manufacturing from a EU company which operates in DK, DE, IT, ES, GB, SW, NO (2011)	
		TiR	1	Ref. year 2008-2011, data from these years	
		C	1	94% of impact categories and 96% of reference flows covered	
		P	2	Data from manufacturing companies based on measure controls and literature (authoritative sources)	
		M	1	Cradle-to-grave, EoL included (recycling, energy recovery, landfilling), allocation not applicable (but allocation by energy and mass used in background system)	
	Ecoinvent	TeR	2	Modelled the onshore and offshore technology mix, but sizes of turbines and capacities are lower than referenced	2.00
		GR	3	Dataset represent an average RER but countries are not well represented	
		TiR	1	Ref. year 2000-2002, data from 1999 and 2001	
		C	1	100% of impact categories and 100% of reference flows covered	
		P	4	Data from manufacturing companies but not possible to check them	
		M	1	Cradle-to-grave, EoL included (recycling, incineration), allocation not applicable	
	GEMIS	TeR	4	Modelled a generic wind farm (10 turbines/1 MW). Not possible to identify the technologies	3.67
		GR	5	A generic dataset cannot be GR for the European context	
		TiR	4	Ref. year 2000, data from 1992-1993	
		C	2	75% of impact categories, 90% of flows covered	
		P	4	Data from manufacturing companies but not possible to check them	
		M	3	Cradle-to-gate, EoL not included, allocation considered but no info	
	E3	TeR	3	Modelled two plants: onshore in Germany, and offshore in Denmark	3.17
		GR	3	Only 2 countries and the installed EU capacity of wind power is not well represented	
		TiR	1	Ref. year 2004, data from 2002-2006	
		C	4	Less than 50% impact categories, 90% flows covered	
		P	3	Info from real plants in DE and DK, technical descriptions are included, but emission factors are not detailed	
		M	5	Cradle-to-gate system, lack of info about EoL and allocation	
Electricity from biomass (DE)	ELCD	TeR	1	Modelled as a technology mix (both electricity and CHP plants)	1.33
		GR	1	Domestic production considered (Germany)	
		TiR	2	Ref. year 2009, some data older than 2005	
		C	1	100% of impact categories and 96% of reference flows covered	
		P	2	Elementary flows from relevant literature (national statistics and official publications), but some of them come from outdated	
		M	1	Cradle-to-grave, EoL included, exergetic and market value allocation	
	GEMIS	TeR	3	Modelled by a generic type of plant	2.33
		GR	1	Domestic production considered (Germany)	
		TiR	2	Ref. year 2010, data from 1989-2005	
		C	2	75% of impact categories, 90% of flows covered	
		P	3	Main data from Oko reports, no info about emission factors or direct emissions	
		M	3	Cradle-to-grave, EoL not included, allocation applied but not defined	
	E3	TeR	3	Modelled by a generic type of plant	3.00
		GR	1	Domestic production considered (Germany)	
		TiR	2	Ref. year 2001, data from 1998-2007	
		C	4	Less than 50% impact categories, 90% flows covered	
		P	3	References come from GEMIS	
		M	5	Cradle-to-gate, EoL not included, allocation not defined but assumed as GEMIS	
Electricity from photovoltaic (DE)	ELCD	TeR	1	Modelled as a technology mix of different PV technologies	1.17
		GR	1	Modelled according a regional specific production in Germany	
		TiR	1	Ref. year 2009, data from 2005-2009	
		C	1	94% of impact categories and 95% of reference flows covered	
		P	1	Data direct from production plants	
		M	2	Cradle-to-grave, EoL not considered, market allocation applied	
	Ecoinvent	TeR	2	Modelled as a technology mix based on worldwide average production	1.33
		GR	2	Modelled considering production processes in US and Europe, but German production. Some correction factors for adapting data to Swiss and German context	
		TiR	1	Ref. year 2007, data from 2002-2007	
		C	1	100% of impact categories and 100% of reference flows covered	
		P	1	Data mainly from production plants or literature (and personal communications)	
		M	1	Cradle-to-grave, EoL included, economic allocation considered	
	GEMIS	TeR	3	Modelled as two types of PV technologies (mono and multi-crystalline)	2.84
		GR	1	Modelled as Europe and US markets. German plants considered	
		TiR	4	Ref. year 2010, data from 1995-2004	
		C	2	75% of impact categories, 90% of flows covered	
		P	3	Data mainly from literature but not possible to check the used data	
		M	3	Cradle-to-gate, EoL not included, allocation applied but not defined	
	E3	TeR	4	Modelled as one type of technology (multi-crystalline)	4.33
		GR	5	Generic power plant, no clear info about its location	
		TiR	4	Ref. year 1992, data from 1995 and 2002 but not possible to check	
		C	4	Less than 50% impact categories, 90% flows covered	
		P	4	Not possible to check references, no info about elementary flows	
		M	5	Not clear the system boundaries and allocation due to lack of info	

## Discussion

The comparison of the selected datasets from different databases, referred to the same technology, can lead to the identification of potential improvements in each quality criteria. Moreover, relevant Authoritative Sources and Business Associations, which could provide additional information to improve the quality of the ELCD results, can be also identified in order to enhance the overall quality of data. It must be remarked that many recommendations are related to future updated versions of ELCD electricity datasets. Table 5 shows a summary of the findings and recommendations that arose from such cross assessment.

**Table 5:** **Recommendations for improving ELCD electricity datasets by DQI**

**ELCD datasets**	**DQI**	**Potential improvements and recommendations**
Electricity grid mix	TeR	• Inclusion of minority technologies could have an important share in the future (e.g. solar thermal technologies already present in the mix of countries like Spain, or ocean technologies or even carbon capture and storage –CCS- technologies).
	C	• To fulfill the criterion in a 100% share, the following flows should be considered: Halon 1211 for ozone depletion, and indium for resource depletion impact category.
	P	• Statistical information used to construct the electricity mixes of each country has been retrieved from the IEA (authoritative source). However, and due to the ELCD database has been developed by the EC in a European context, it seems adequate to use the data reported by each country to Eurostat.
	M	• Inclusion of the EoL modelling of PV facilities.
	General	• In order to have a more useful database in which users can update the EU27 electricity mix, datasets not only by country but also by technology should be available.
		• Some analysed databases make use of energy models to derive future European electricity mixes, although this is not the scope of the ELCD.
Electricity from fossil fuels (hard coal, lignite and natural gas)	TeR	• CCS technologies can be included due to the importance in future environmental scenarios, as stated in several studies (Koornneef et al., 2008; Stanley and Dávila-Serrano., 2012).
		• Several future clean coal, lignite and natural gas electricity scenarios can be developed and included in the ELCD datasets, as another database (GEMIS) includes.
	C	• To fulfill the criterion in a 100% share, the following flows should be considered: Halon 1211 for ozone depletion, and indium for resource depletion impact category.
	P	• The use of a database with well reported emissions based on data from a large power plant database in Europe, such as in Ecoinvent, could improve the results.
		• Some Business Associations publications can be useful for compiling precise and updated inventories: European Association of Coal and Lignite (Euracoal, http://www.euracoal.be), the Union of Electricity Industry (Eurelectric, http://www.eurelectric.org) and the European Association of Gas Wholesale, Retail and Distribution Sectors (Eurogas, http://www.eurogas.be), the Gas Infrastructure Europe (http://www.gie.eu.com), the Transmission System Operators, Storage Systems Operator and Terminal Operators, and the Technical Association of the European Natural Gas Industry (MARCOGAZ, http://www.marcogaz.org).
Electricity from nuclear power (FR and DE)	GR	• It could be improved using data from Canadian mines and mills that can be obtained from CERI (2008) or UNSCEAR (1993, 2000). In case of the French dataset, conversion data in facilities are available in the ExternE study of the French nuclear fuel cycle (EC, 1995).
	TiR	• In both German and French datasets, TiR is the worst scored category in the ELCD database. The reason lies on the use of several old references. However, no better references could be found in the other databases analysed in this study. Other datasets (e.g. Ecoinvent) perform better since the validity period of the dataset is closer to the oldest references.
	P	• Concerning radioactive emissions data, uncertainty can be decreased by using data published by UNSCEAR (2000), considered as an authoritative source.
	M	• It could be improved with the consideration of a final repository for spent fuel and high activity waste. Data source can be those included in NAGRA (2002a, 2002b).
Electricity from hydro power	TeR	• In a future scenario, Small Hydropower Plants (SHPP) should be included due to the potential importance in the mix. According to statistical data from Arcadis (2011), a considerably reduction of electricity from hydropower mix is expected and the large facilities might be the main affected. Then, the share of SHPP in electricity from hydropower mix might increase; although a reduction of their potential is foreseen.
		• To get additional inventory data, ESHA (European Small Hydropower Association, http://www.esha.be) publishes EU data facts and statistics of hydropower generation.
	P	• The inclusion of documentation related to the data collection process and additional references to identify the origin of the data values could be useful to achieve a better rating. On the other hand, the IHA (International Hydropower Association, http://www.hydropower.org/), might be a relevant information source for double checking (annual reports and GHG Risk Assessment Tool that provides estimation of the level of gross GHG emissions from freshwater reservoir).
	C	• It can be fulfilled completely with the consideration of Halon 1211 for ozone depletion, and cadmium and indium for resource depletion impact category. It must be highlighted that ELCD includes the emissions due to biomass degradation, while other datasets do not consider them.
Electricity from wind power	TeR	• Capacity factors and average sizes described are in line with the statistics provided by authoritative sources, such as the European Wind Energy Association (EWEA) and the International Energy Agency (IEA). It would be recommended to include additional documentation, providing more detail concerning the different shares of onshore and offshore power as well as the contribution of each country to the total mix (e.g. the British Wind Energy Association). Additionally, it is recommended to review for future versions other wind options, such as the small and medium scale wind, which might increase in the future, and the re-powering, which substitutes old turbines, increasing the capacity.
	GR	• ELCD dataset models a non-defined region in Europe. It must be highlighted that this energy source has a very site-specific resource and therefore, this technology applied in each European country and their contribution to the total electricity generation by wind in Europe might vary. However, ELCD takes into account this particularity by considering the full load hours for the actual region using statistical information.
	C	• It could be 100% fulfilled with the inclusion of Halon 1211 and CFC-12 for ozone depletion and indium for resource depletion impact category.
	P	• The Wind Power Net (http://www.thewindpower.net) gives access to a large database with the current commercial wind turbines and the installed wind farms in the world. It provides information about the location of the farm, technology use, type of turbine, capacities, etc. This database can be used for double check some data.
	M	• If re-powering systems are to be included in future versions, other EoL scenarios should be reviewed and considered, if applicable.
Electricity from biomass (DE)	C	• In order to improve the criterion, Halon 1211 for ozone depletion, and cadmium and indium for resource depletion impact category, should be considered.
	General	• If this German dataset is going to be used for other European conditions, the scores would be much lower. Results, especially from the forestry module, cannot be extrapolated to the European conditions since forestry management activities are very variable across Europe. The dataset should be split in several ones representing other forestry management practices and yields such us Nordic or Mediterranean countries forestry (nevertheless, updated versions of ELCD include dataset for different regions).
		• It should be noted that no additional authoritative source has been found that could improve the ELCD dataset.
Electricity from photovoltaic	TeR	• It should be noted that this dataset has been modelled in a way that the European current technology is included. Among the other databases, the ELCD dataset contains the most updated information and provides deep details concerning the precision of the data used. To model this technology at least two relevant Authoritative Bodies have been used: the European Photovoltaic Technology Platform (EPTP) and the EurObserv’ER Barometer (http://www.eurobserv-er.org). The European Photovoltaic Industry Association (http://www.epia.org) provides detailed information related to the evolution of this sector yearly, and should be considered a relevant source for future versions.
	C	• In order to improve the criterion, CFC-14 for climate change, Halon 1211 for ozone depletion, and indium for resource depletion impact category, should be also considered.
	M	• ELCD should include also an EoL scenario in future versions (e.g from Lozanovski & Held, 2010). Moreover, it can be improved with the inclusion of a basic scenario of dismantling and waste treatment, considering main materials, such as steel or plastics, as Ecoinvent (2012).

## Conclusions and recommendations

This extended analysis of the ELCD electricity datasets aimed at providing better founded information related to its data quality, following the indicators developed and described within the ILCD handbook (EC-JRC, 2011). This analysis, together with the study on ELCD fuel datasets (Garraín et al. Background qualitative analysis of the European Reference Life Cycle Database (ELCD) energy datasets – Part I: Fuel datasets. Springer Plus - Submitted in 2014), have meant an opportunity to implement these quality indicators to different datasets for the first time. It has had two main consequences. Firstly, the implementation of the quality indicators to the energy-related datasets from the ELCD has been used to understand the room for improvement in future ELCD versions. Additionally, it has also served to identify whether these data quality indicators are applicable and useful for the database developers in general, as well as for the LCA practitioners. It should be stated that results obtained from this analysis ensure the quality of the energy-related datasets to any LCA practitioner, and provide insights related to the limitations and assumptions underlying in the datasets modelling. Giving this information, the LCA practitioner will be able to decide whether the use of the ELCD datasets is appropriate based on the goal and scope of the analysis to be conducted.

Along the current analysis, several assumptions have been made in order to facilitate the analysis, such as the selection of databases and datasets or the definition of data quality indicators (DQIs). The results have to be understood under this context. Taking those considerations into account, the data quality assessment conducted in here should not be extrapolated to datasets under different contexts. Furthermore, the analysis has been performed only in a selection of the most representative electricity datasets from the ELCD as well as from the other selected databases. The conclusions obtained in this analysis cannot be extrapolated to other type of datasets, nor can be used to compare databases among them.

From the deep analysis conducted, it must be highlighted that the ELCD datasets have been modelled based on an extensive review of the most relevant literature and statistics. The documentation used to model the ELCD energy related datasets can be found in the Life Cycle Thinking Platform web-site (http://eplca.jrc.ec.europa.eu/ELCD3/).

In terms of the quality criteria, the analysed ELCD datasets showed a very good performance in the majority of the criteria, especially in those criteria related to TeR, C and M. Nevertheless, several recommendations for improving have been detailed above.

Concerning the different technologies analysed, ELCD datasets have the best quality rating in the majority of the technologies, with the exception of electricity from nuclear datasets in which TiR and M criteria score worse than other databases and PV dataset where M criterion also performs worse than in other databases. Several recommendations have been also made to overcome these limitations.

One of the most relevant weaknesses of the ELCD is the lack of datasets that model electricity produced by each technology in each European country. Currently, the ELCD includes electricity mix datasets for each country, modelled considering an established share of sources that might be different to the needs of the user. The inclusion in future versions will improve the flexibility and usefulness of the database. Moreover, in some renewable technologies (PV or biomass) regional specificities are not always well considered in terms of capacity factors, forest management, etc. In these cases, it would be desirable to split the dataset in different country specific or bioclimatic regions datasets. Although the optimal solution to this limitation would be to model new datasets for electricity production by technology and for each country, this might not be feasible in the short term. An alternative solution would be to model datasets for each technology under a European context, and to introduce parameters in the electricity mix datasets to vary the shares of each technology. In order to give response to any change or advance in technologies, and to be able to model new datasets and/or to modify the current ones if necessary, it is highly recommended to constantly review the evolution of advanced technologies and their share in the European market. According to the approach endorsed by several authors for the modelling of consequential-LCA of energy systems (see e.g. Igos et al. 2014, Vázquez-Rowe et al. 2014, Earles et al. 2013, and Dandres et al. 2012), also future electricity scenarios can be developed using data from energy projective models such as PRIMES or TIMES (The Integrated MARKAL-EFOM System). This is an important improvement of the database that could be very useful for prospective and consequential LCA studies.

Modelling the End of Life (EoL) of the systems appears to be a difficult task due to the novelty of some technologies and the lack of data from other technologies (solar PV, final repository for spent nuclear fuel and natural gas plant dismantling). Efforts on this challenge should be kept in the future.

Regarding the use of authoritative sources, the ELCD database makes extensive use of the statistical information provided by the IEA. This is of course an authoritative source. However, for the European context it seems appropriates the use of data reported by each country to Eurostat. In order to improve precision, it would be advisable to make a more extensive use of Business Associations and Authoritative sources data that have been proposed throughout the analysis.

Since its first release, the ELCD database has been updated two times. The needs of reviewing and updating the ELCD database depend on the different sectors and the technologies. It would be useful to define periods to revise the electricity related datasets. For this purpose, a deep analysis of the learning curves would identify the level of maturity for each technology. Then, special periods for reviewing could be identified by technology.

Finally, it should be noted that the selected databases are in a constant process of updating and improvement, *e.g.* Ecoinvent v3.0 or GEMIS v4.93, so a detailed analysis of these, together with a deeper analysis of related reference documents (Treyer and Bauer, 2013, 2014) can offer further potential improvements to future ELCD versions.
